# Development and validation of a predictive model for diagnosing prostate cancer after transperineal prostate biopsy

**DOI:** 10.3389/fonc.2022.1038177

**Published:** 2022-12-01

**Authors:** Wenming Ren, Yujie Xu, Congcong Yang, Li Cheng, Peng Yao, Shimin Fu, Jie Han, Dong Zhuo

**Affiliations:** Department of Urology, The First Affiliated Hospital of Wannan Medical College, Wuhu, Anhui, China

**Keywords:** biopsy, biomarkers, nomogram, diagnosis, prostate cancer

## Abstract

**Objective:**

This study aimed to develop and validate a nomogram to predict the probability of prostate cancer (PCa) after transperineal prostate biopsy by combining patient clinical information and biomarkers.

**Methods:**

First, we retrospectively collected the clinicopathologic data from 475 patients who underwent prostate biopsy at our hospital between January 2019 to August 2021. Univariate and multivariate logistic regression analyses were used to select risk factors. Then, we established the nomogram prediction model based on the risk factors. The model performance was assessed by receiver operating characteristic (ROC) curves, calibration plots and the Hosmer–Lemeshow test. Decision curve analysis (DCA) was used to evaluate the net benefit of the model at different threshold probabilities. The model was validated in an independent cohort of 197 patients between September 2021 and June 2022.

**Results:**

The univariate and multivariate logistic regression analyses based on the development cohort indicated that the model should include the following factors: age (OR = 1.056, *p* = 0.001), NEUT (OR = 0.787, *p* = 0.008), HPR (OR = 0.139, *p* < 0.001), free/total (f/T) PSA (OR = 0.013, *p* = 0.015), and PI-RADS (OR = 3.356, *p* < 0.001). The calibration curve revealed great agreement. The internal nomogram validation showed that the C-index was 0.851 (95% CI 0.809-0.894). Additionally, the AUC was 0.851 (95% CI 0.809-0.894), and the Hosmer–Lemeshow test result presented *p* = 0.143 > 0.05. Finally, according to decision curve analysis, the model was clinically beneficial.

**Conclusion:**

Herein, we provided a nomogram combining patients’ clinical data with biomarkers to help diagnose prostate cancers.

## Introduction

Prostate cancer (PCa) has the second highest incidence of all malignant tumors in men worldwide and ranks first in the incidence of male tumors in more than half of countries. Additionally, the mortality rate of PCa is the fifth highest among male cancers (1).PCa is a highly heterogeneous tumor. Cases with higher Gleason score (≥7), defined as clinically significant PCa (csPCa), usually show a high aggressiveness and a tendency for rapid progression. In contrast, the PCa patients with lower Gleason score progressed slowly (2, 3).Prostate-specific antigen (PSA) is the most used early detection marker for prostate cancer. However, the PSA specificity is weak (20-40%), and other conditions, such as benign prostatic hyperplasia (BPH), can affect PSA levels (4). Prostate biopsy is now the standard for prostate cancer diagnosis (5). Meanwhile, biopsy is an invasive operation, and systematic biopsy often shows false negative results. Besides, increasing the puncture points can lead to complications such as bleeding, urinary retention and infection (6).

Therefore, PCa screening based on PSA level as the sole indication for prostate biopsy lacks specificity and may lead to unnecessary biopsy. As such, clinical practice urgently needs new methods for early, noninvasive screening of prostate cancer. In recent years, several blood biomarkers (2, 3, 7–9), urine biomarkers (2, 3, 7, 10, 11) and multiparametric magnetic resonance imaging (mpMRI) (11, 12) have been developed to predict PCa. Several of PSA derivatives, especially free PSA/TPSA (f/TPSA), and PSA density (PSAD) have been demonstrated as promising biomarkers (8, 9). Urine metabolomics in the early detection, risk phase, and treatment prognosis of prostate cancer studies have been reported (2, 3, 7, 10, 11). mpMRI has also been validated as a reliable radiological technique for prostate cancer diagnosis, targeted biopsy, tumor staging, and monitoring (11, 12).

Current studies have shown that tumorigenesis and development are tightly linked with inflammation, and neutrophils are associated with the occurrence and development of human cancers, including lung (13) and breast (14) cancers. Additionally, the clinical significance of the hemoglobin to platelet ratio (HPR) has been demonstrated in colon cancer (15) and renal cell carcinoma (16). Furthermore, the clinical significance of neutrophil to lymphocyte ratio (NLR), platelet to lymphocyte ratio(PLR),monocyte-to-lymphocyte ratio (MLR) and systemic immune-inflammation index (SII) has also been confirmed (17, 18).

Therefore, in the present study, we developed and validated a PCa prediction model combining inflammatory biomarkers with the clinical data of patients. Based on our current results, the model can be used to assist in the screening of PCa.

## Materials and methods

### Patients’ data

**Figure 1:**
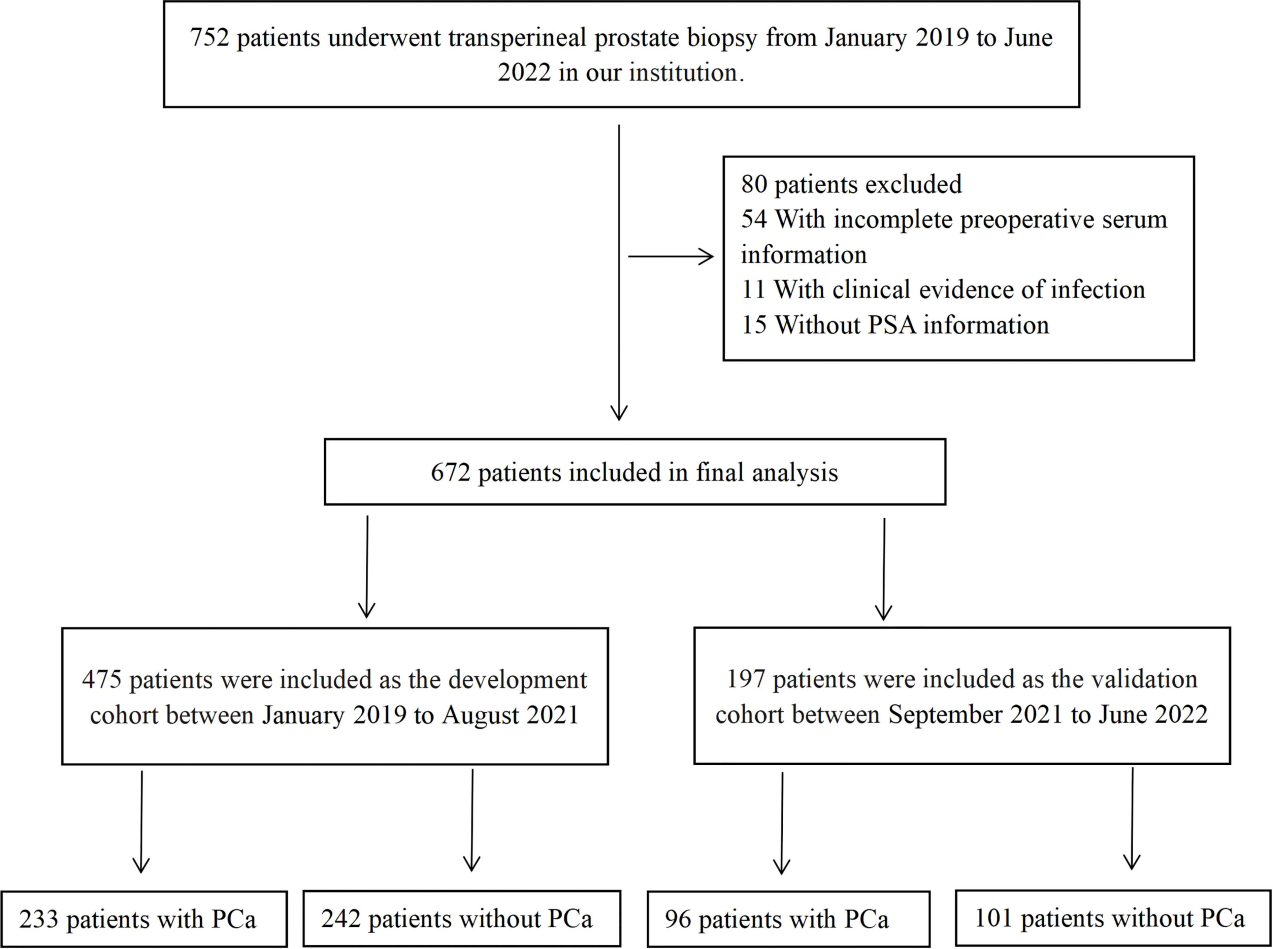
The flowchart of patients enrolled in this study. PSA, prostate specific antigen; PCa, prostate cancer.

First, 752 patients who underwent transperineal prostate biopsy in our institution from January 2019 to June 2022 were enrolled, and 672 patients were finally included. The flowchart of patients enrolled is shown in **Figure 1**. Then, patients were separated into two groups according to the date of their prostate biopsy: development cohort: 475 patients who underwent prostate biopsy at our center between January 2019 to August 2021; validation cohort: 197 consecutive patients who received the same operation from September 2021 and June 2022.The pathological results of each patient were determined by the same pathologist based on the 8^th^ American Joint Committee on Cancer (AJCC).

The inclusion criteria were as follows: man with abnormal PSA levels

(PSA>4ng/ml) or with suspicious lesions on imaging(PI-RADS score≥3) were recruited. The population included first-time biopsy and those with previous negative biopsy. All patients underwent MRI before biopsy, and prostate biopsy performed *via* ultrasound guidance. The interval between MRI and biopsy was less than 15 days.

### Variables

Demographic and laboratory test results were retrospectively retrieved from our medical system. The HPR was calculated as the hemoglobin to platelet ratio. NLR was defined as neutrophil count divided by lymphocyte count, PLR was defined as platelet count divided by lymphocyte count, SII= (neutrophil × platelet)/lymphocyte. MLR was calculated as monocyte-to-lymphocyte count. The prostate volume (PV) was estimated with the MRI‐based modified ellipsoid formula: 0.523*(max width × max length × max height). The PSA density (PSAD) was calculated by dividing the TPSA level by the PV. f/T (calculated as the fPSA to TPSA ratio). The PI‐RADS score (1–5) was evaluated by specialists based on the T2WI, DWI, and DCE, according to the Prostate Imaging-Reporting and Data System version 2 (PI‐RADS v. 2).

### Biopsy methods

Patients who underwent prostate biopsy should present PSA > 10 ng/ml or PSA 4-10 ng/ml combined with suspicious lesions on imaging(PI-RADS score≥3) or normal PSA level combined with imaging suspicious lesions(PI-RADS score≥3). Some patients underwent a second biopsy because their repeat PSA suggested suspicious prostate cancer. All operations were carried out by the same specialist doctor at our institute. Under local anesthetic, a systematic 13-core or 3 (Targeted) + 12 (systematic)-core prostate biopsy was performed *via* ultrasound guidance.

### Statistical analysis

Data were analyzed using SPSS 23.0 statistical software (IBM SPSS INC., Chicago, USA) and R software (v. 4.1.3 - Institute of Statistics and Mathematics, Vienna, Austria).The reported statistical significance levels were two-sided, with p<0.05.Continuous variables with normal distribution were presented as means ± standard deviations (SD) and were analyzed by independent sample *t*-tests. Non‐normal continuous variables were presented as medians (interquartile ranges) and were analyzed by the Mann–Whitney U-test. Categorical variables were presented as numbers (percentages). The correlations between categorical variables were analyzed using Pearson’s or Continuity Corrected χ^2^-test.

Univariate logistic regression analysis was used to investigate independent risk factors(*P*<0.05) associated with PCa in the development cohort. Then, a multivariate logistic regression analysis was performed for all significant risk variables, using backward stepwise regression to select prostate cancer risk predictors(*P*<0.05).A nomogram prediction model was established based on the risk factors selected by multivariate analysis.

The discrimination performance of the model was assessed by the area under the receiver operator characteristic curve (AUC). Calibration of the nomogram was evaluated using calibration plots (bootstrap method, 1000 repetitions) and Hosmer-Lemeshow test (P>0.05 indicates good agreement). The DCA curve was used to evaluate the net benefit ratio of the model at different probability thresholds. The performance of the nomogram was tested in an independent validation cohort divided by time.

## Results

### Baseline characteristics

A total of 475 patients were enrolled in the development cohort, and 197 were included in the validation cohort. The demographic and clinicopathological data of patients were presented in **Table 1**. A total of 233 (49.05%) patients in the development cohort and 96 (48.73%) in the validation cohort had PCa. Compared to the non-PCa group, the PCa group had a lower preoperative NEUT(neutrophils), HGB(hemoglobin), higher PLT(platelet), and lower HPR (**Supplementary Figure 1**).

**Table 1:** Characteristics and blood biomarkers of patients in this study.

Variable	Overall(n=672)			Development Cohort(n=475)			Validation Cohort(n=197)		
	PCa group n=329	non PCa group n=343	P value	PCa group n=233	non PCa group n=242	P value	PCa group n=96	non PCa group n=101	P value
Age (years)	71.64±8.48	66.89±8.38	<0.001	71.19±8.66	66.62±8.60	<0.001	72.74±7.94	67.53±7.83	<0.001
Hypertension						0.678			0.044
No	149 (22.2)	165 (24.5)	0.465	116 (49.8)	116 (47.9)		33 (16.7)	49 (24.9)	
Yes	180 (26.8)	178 (26.5)		117 (50.2)	126 (52.1)		63 (32.0)	52 (26.4)	
Diabetes mellitus			0.173			0.095			0.938
No	281 (41.8)	305 (45.4)		197 (84.5)	217 (89.7)		84 (42.6)	88 (44.7)	
Yes	48 (7.1)	38 (5.7)		36 (15.5)	25 (10.3)		12 (6.1)	13 (6.6)	
Coronary heart disease			<0.001			0.002			0.006
No	263 (39.1)	312		188 (80.7)	219 (90.5)		75 (38.1)	93 (47.2)	
Yes	66 (9.8)	31		45 (19.3)	23 (9.5)		21 (10.6)	8 (4.1)	
BMI (kg/m2)	23.01±3.31	23.25±2.96	0.311	22.86±3.24	23.17±2.90	0.278	23.37±3.47	23.46±3.08	0.842
ASA score 1/2/3/4	2.00 (2.00,3.00)	1.00 (1.00,2.00)	<0.001	2.00 (2.00,3.00)	2.00 (1.00,3.00)	<0.001	2.00 (2.00,3.00)	2.00 (1.00,2.00)	<0.001
Hematuria			0.269			0.323			0.592
No	306 (45.5)	311 (46.3)		220 (94.4)	223 (92.1)		86 (43.7)	88 (44.7)	
Yes	23 (3.4)	32 (4.8)		13 (5.6)	19 (7.9)		10 (5.1)	13 (6.5)	
History of biospy			0.467			0.347			1.000
No	319 (47.5)	329 (48.9)		227 (97.4)	232 (95.9)		92 (46.7)	97 (49.2)	
Yes	10 (1.5)	14 (2.1)		6 (2.6)	10 (4.1)		4 (2.0)	4 (2.0)	
History of prostate surgery			0.784			0.711			1.000
No	316 (47.0)	328 (48.9)		224 (96.1)	231 (95.5)		92 (46.7)	97 (49.2)	
Yes	13 (1.9)	15 (2.2)		9 (3.9)	11 (4.5)		4 (2.0)	4 (2.0)	
Family history of PCa			0.956			0.966			1.000
No	325	340		230	240		95	100	
Yes	4	3		3	2		1	1	
NEUT (10^9^/L)	3.87±1.36	4.42±1.81	<0.001	3.89±1.44	4.30±1.67	0.005	3.83±1.17	4.72±2.08	<0.001
LYM (109/L)	1.67±0.61	1.75±0.61	0.099	1.66±0.59	1.73±0.60	0.196	0.65±0.07	0.63±0.06	0.306
MO (10^9^/L)	0.43±0.18	0.45±0.16	0.171	0.43±0.14	0.45±0.16	0.150	0.24±0.02	0.17±0.02	0.013
HGB (g/L)	130.90±19.65	137.46±15.35	<0.001	131.03±20.49	135.96±16.19	0.004	130.60±17.53	141.04±12.46	<0.001
PLT (10^9^/L)	191.29±59.39	166.73±49.68	<0.001	190.01±59.31	166.70±54.67	<0.001	194.20±59.80	166.82±35.18	<0.001
NLR	2.61±1.66	3.04±3.40	0.036	2.64±1.84	2.83±1.74	0.256	1.15±0.12	5.64±0.56	0.077
PLR	127.17±56.33	107.12±59.25	<0.001	112.80±49.33	118.64±51.62	0.208	59.57±6.08	80.33±7.99	0.065
SII	503.58±398.30	496.66±514.78	0.846	509.94±437.82	462.97±286.18	0.169	281.56±28.74	836.42±83.23	0.322
MLR	0.28±0.15	0.29±0.22	0.532	0.28±0.11	0.29±0.14	0.478	0.21±0.02	0.34±0.03	0.784
HPR	0.74±0.23	0.91±0.32	<0.001	0.75±0.23	0.91±0.35	<0.001	0.73±0.22	0.89±0.22	<0.001
TPSA (ng/ml)	15.22 (10.07,35.1)	10.99 (7.71,16.6)	<0.001	15.34 (9.57,37.00)	11.3 (7.85,17.77)	<0.001	16.50 (10.97,43.68)	10.30 (7.03,15.25)	<0.001
fPSA (ng/ml)	2.25 (1.21,4.31)	1.90 (1.16,2.80)	0.001	2.63 (1.32,5.09)	2.11 (1.29,3.19)	0.022	1.90 (1.09,5.72)	1.61 (1.12,2.40)	0.023
f/T	0.13 (0.09,0.19)	0.18 (0.12,0.23)	<0.001	0.14 (0.09,0.20)	0.19 (0.13,0.25)	<0.001	0.11 (0.08,0.15)	0.16 (0.11,0.21)	<0.001
PV (ml)	41.44 (27.27,63.03)	54.76 (40.79,77.72)	<0.001	44.04 (29.13,68.10)	49.81 (37.09,70.98)	0.015	41.80 (26.95,59.87)	58.70 (45.24,82.21)	<0.001
PASD (ng/ml/ml)	0.43 (0.21,0.85)	0.19 (0.12,0.33)	<0.001	0.43 (0.20,0.85)	0.21 (0.14,0.38)	<0.001	0.46 (0.24,0.91)	0.15 (0.11,0.24)	<0.001
PI-RADS score 1/2/3/4/5	4.00 (3.00,5.00)	3.00 (2.00,3.00)	<0.001	4.00 (4.00,5.00)	3.00 (2.00,4.00)	<0.001	4.00 (3.00,4.00)	2.00 (2.00,3.00)	<0.001

PCa, prostate cancer; BMI, body mass index; ASA, American Society of Anesthesiologists; NEUT, neutrophils; LYM, Lymphocyte; MO, Monocyte; HGB, hemoglobin; PLT, platelet; NLR, neutrophil to lymphocyte ratio; PLR, platelet to lymphocyte ratio; SII, systemic immune-inflammation index; MLR, monocyte-to-lymphocyte ratio;HPR, hemoglobin-platelet ratio; PSA, prostate-specific antigen; fPSA, free prostate-specific antigen; f/T, fPSA/ TPSA; PV, prostate volume; PSAD, prostate specific antigen density; PI-RADS, Prostate Imaging Reporting and Data System.

In the development cohort, PCa patients were older (71.19 ± 8.66 years) than the control group (66.62 ± 8.60 years). The average TPSA, fPSA, and PSAD of PCa patients were higher compared to non-PCa patients. The f/T distribution was as follows: 0.14 (0.09-0.20) for PCa patients and 0.19 (0.13-0.25) non-PCa patients. Moreover, the distribution of coronary heart disease, ASA score, and PI-RADS scores significantly differed between the two groups. In the validation cohort, the differences between the groups of variables were consistent with the development cohort (**Table 1**).

### Logistic regression analysis of clinical features and biomarkers

Further, the logistic regression analysis was used to identify the independent risk factors to predict PCa in the development cohort. The univariate analysis revealed that 11 variables were significantly associated with PCa: age, coronary heart disease, ASA score, NEUT, HGB, PLT, PLR, HPR, TPSA, fPSA, f/T, PASD, and PI-RADS score (*p <* 0.05) (**Table 2**).

**Table 2:** Univariable analysis of patients in the development and validation cohorts.

Variable	Development Cohort				Validation Cohort			
	β	OR	95%CI	P value	β	OR	95%CI	P value
Age	0.062	1.064	(1.041,1.089)	<0.001	0.086	1.090	(1.047,1.135)	<0.001
Hypertension	-0.074	0.929	(0.648,1.331)	0.622	0.587	1.799	(1.013,3.194)	0.045
Diabetes mellitus	0.461	1.586	(0.919,2.737)	0.097	-0.034	0.967	(0.418,2.239)	0.938
Coronary heart disease	0.824	2.279	(1.330,3.907)	0.003	1.18	3.255	(1.365,7.764)	0.008
BMI	-0.033	0.968	(0.912,1.027)	0.276	-0.009	0.991	(0.910,1.080)	0.841
ASA score	0.54	1.716	(1.352,2.178)	<0.001	0.848	2.334	(1.461,3.728)	<0.001
Hematuria	-0.366	0.694	(0.334,1.439)	0.326	-0.239	0.787	(0.328,1.891)	0.592
History of biopsy	-0.489	0.613	(0.219,1.715)	0.351	0.053	1.054	(0.256,4.340)	0.942
History of prostate surgery	-0.170	0.844	(0.343,2.075)	0.711	0.053	1.054	(0.256,4.340)	0.942
Family history of PCa	0.448	1.565	(0.259,9.453)	0.625	0.051	1.053	(0.065,17.069)	0.971
NEUT	-0.174	0.840	(0.743,0.950)	0.005	-0.367	0.693	(0.558,0.860)	0.001
LYM	-0.202	0.817	(0.601,1.110)	0.197	-0.232	0.793	(0.509,1.235)	0.305
MO	-0.882	0.414	(0.124,1.378)	0.151	-0.300	0.740	(0.189,2.899)	0.666
HGB	-0.015	0.985	(0.975,0.995)	0.004	-0.048	0.953	(0.932,0.974)	<0.001
PLT	0.007	1.008	(1.004,1.011)	<0.001	0.014	1.014	(1.007,1.021)	<0.001
NLR	-0.061	0.94	(0.844,1.048)	0.265	-0.135	0.874	(0.724,1.054)	0.158
PLR	-0.002	0.998	(0.994,1.001)	0.209	0.004	1.004	(0.999,1.009)	0.083
SII	0.0004	1.000	(1.000,1.001)	0.181	0.0003	1.000	(0.999,1.000)	0.346
MLR	-0.513	0.599	(0.144,2.482)	0.479	-0.142	0.868	(0.315,2.391)	0.784
HPR	-2.039	0.130	(0.064,0.266)	<0.001	-3.527	0.029	(0.007,0.131)	<0.001
TPSA	0.023	1.024	(1.012,1.035)	<0.001	0.053	1.054	(1.027,1.082)	<0.001
fPSA	0.057	1.058	(1.011,1.108)	0.015	0.248	1.281	(1.103,1.488)	0.001
f/T	-5.297	0.005	(0,0.065)	<0.001	-8.819	0.001	(0,0.024)	0.001
PV	0.0004	1.000	(0.995,1.004)	0.872	-0.022	0.979	(0.968,0.990)	0.979
PASD	0.895	2.447	(1.576,3.799)	<0.001	3.221	25.047	(6.388,98.213)	<0.001
PI-RADS score	1.243	3.467	(2.690,4.468)	<0.001	1.139	3.124	(2.156,4.524)	<0.001

PCa, prostate cancer; BMI, body mass index; ASA, American Society of Anesthesiologists; NEUT,neutrophils;

LYM,Lymphocyte;MO,Monocyte; HGB, hemoglobin; PLT, platelet;NLR, neutrophil to lymphocyte ratio; PLR, platelet to lymphocyte ratio;SII, systemic immune-inflammation index;MLR, monocyte-to-lymphocyte ratio;HPR, hemoglobin-platelet ratio;

PSA, prostate-specific antigen; fPSA, free prostate-specific antigen; f/T, fPSA/ TPSA; PV, prostate volume; PSAD, prostate specific antigen density; PI-RADS, Prostate Imaging Reporting and Data System. Red values means P value <0.005.

Next, the multivariate analysis showed that NEUT (OR = 0.787, 95% CI: 0.658-0.941, *p* = 0.008), HPR (OR = 0.139, 95% CI: 0.047-0.417, *p* < 0.001), and f/T (OR = 0.013, 95% CI: 0-0.426, *p* = 0.015) were independent protective factors for PCa. Besides, our current results indicated that age (older) was also an independent risk factor for PCa (OR = 1.056, 95% CI: 1.022-1.092, *p* = 0.001), as well as a higher PI-RADS score (OR = 3.356, 95% CI: 2.445-4.606, *p ≤* 0.001) (**Table 3** and **Supplementary Figure 2**).

**Table 3:** Multivariable analysis of patients in the development and validation cohorts.

Variable	Development Cohort				Validation Cohort			
	β	OR	95%CI	P value	β	OR	95%CI	P value
Age	0.055	1.056	(1.022,1.092)	0.001	0.095	1.100	(1.041,1.163)	0.001
NEUT	-0.240	0.787	(0.658,0.941)	0.008	-0.762	0.468	(0.305,0.716)	<0.001
HPR	-1.971	0.139	(0.047,0.417)	<0.001	-3.088	0.046	(0.005,0.405)	0.006
f/T	-4.328	0.013	(0,0.426)	0.015	-10.359	0.001	(0,0.046)	0.005
PI-RADS score	1.211	3.356	(2.445,4.606)	<0.001	1.224	3.401	(2.075,5.573)	<0.001

NEUT, neutrophils;HPR, hemoglobin-platelet ratio;f/T, fPSA/ TPSA; PI-RADS, Prostate Imaging Reporting and Data System. Red values means P value <0.005.

### Nomogram for PCa prediction

**Figure 2:**
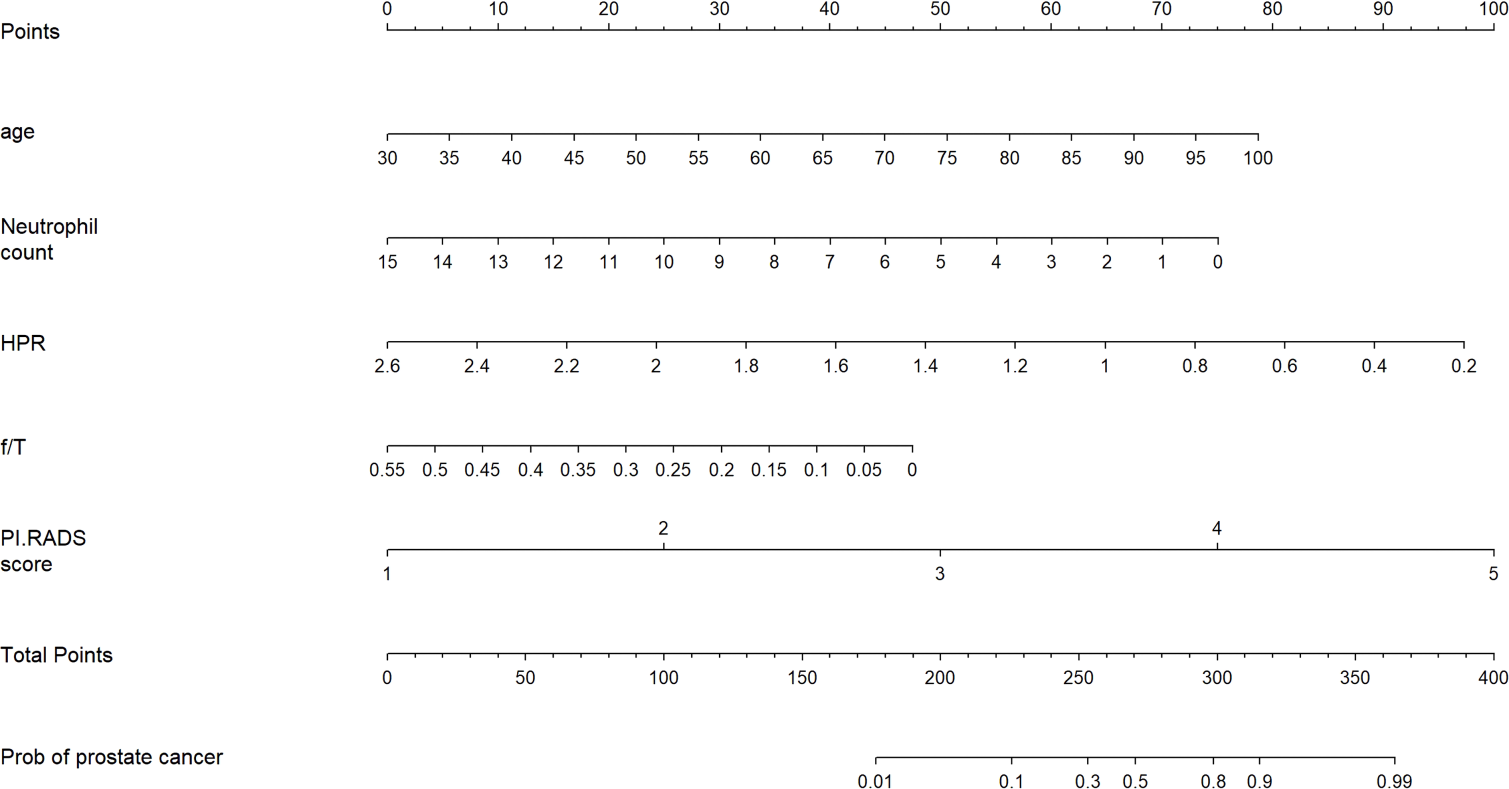
Nomogram for predicting the probability of prostate cancer in patients after transperineal prostate biopsy.

Based on the multivariate analysis, the nomogram included age, NEUT, HPR, f/T, and PI-RADS scores (**Table 3** and **Figure 2**). In the nomogram, each clinical feature corresponds to a particular point. The score corresponding to this point was found on the “Points” axis, and the individual scores were added together to calculate the total score. On the “Prob of prostate cancer” axis, the probability corresponding to the point on the “Total Points” axis comprehends the probability of a patient having PCa.

**Figure 3:**
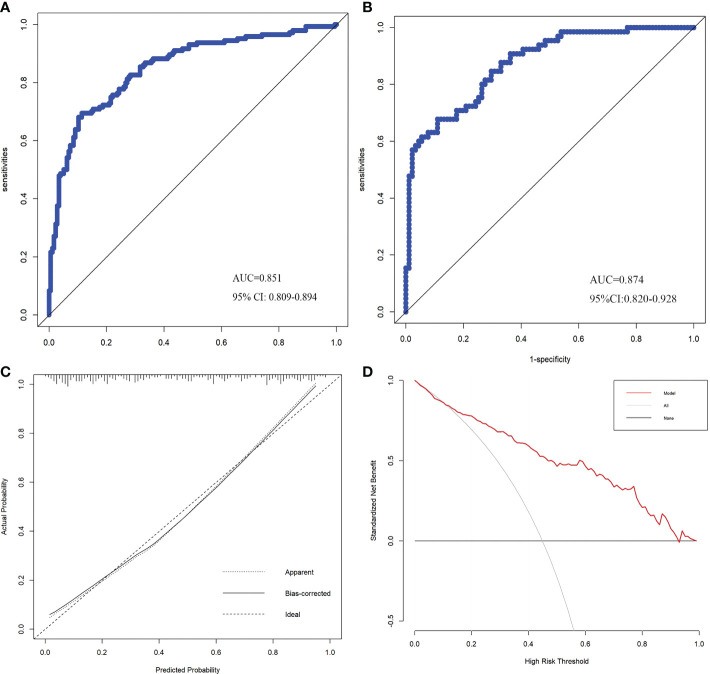
ROC curves of the development **(A)** and validation **(B)** cohorts. Calibration curve of the prediction model **(C)**. Decision Curve Analysis curve of the prediction model **(D)**.

The internal validation of the nomogram showed that the C-index was 0.851 (95% CI: 0.809-0.894). The AUC was 0.851 (95% CI: 0.809-0.894) for the development cohort and 0.874 (95% CI: 0.820-0.928) for the validation cohorts (**Figures 3A, B**). The calibration curve presented great agreement (**Figure 3C**). Additionally, the Hosmer–Lemeshow test showed χ^2^ = 2.15 and *p* = 0.143. These results demonstrated that the nomogram model could predict PCa risk and was greatly consistent with the real risk. According to decision curve analysis, patients with a 10 to 90% threshold probability will benefit from adopting this prediction model for PCa after biopsy (**Figure 3D**).

## Discussion

PSA and its derivatives are widely used in PCa detection, including free PSA/TPSA (f/TPSA), PSA density (PSAD) and precursor forms of PSA (3, 8, 9, 19, 20). The f/TPSA is one of the early diagnostic tools of prostate cancer (9). In patients with a PSA range between 2.5 – 10ng/ml, f/T PSA <10% is an important risk factor for prostate cancer (19). In a Chinese patient-based study, f/T PSA was better than PSA in patients with predicted PSA of 4.0 – 10.0ng/ml. For those age> 60 years, the PSA range was adjusted to 10-20ng/ml (20). A study of PSA density (PSAD) showed that PSA density was significantly better than PSA in distinguishing intraprostatic inflammation from clinically meaningful PCa (csPCa). And this study further showed that in patients with PSA> 4 ng/ml, the PSA density of diagnosed csPCa is >0.15 ng/ml ^2^ and > 0.10 ng/ml ^2^, respectively (8). In addition, other blood markers have also been tested clinically, including the prostate health index (PHI) test and four-kallikrein score (4Kscore). The prostate Health Index (PHI) test including free and total PSA and the [–2]pro-PSA isoform (p2PSA).The four kallikrein (4K) score including free, intact and total PSA and kallikrein-like peptidase 2 (hK2).They have great accuracy in the preliminary diagnosis and prediction of csPCa (2, 7). In this study, we included four PSA related indicators, include PSA, fPSA, free/total PSA ratio, and PSAD. Although they were significant association with prostate cancer (all *p*<0.05) according to the univariate analysis. However, only f/T PSA was significant in multivariate analysis (OR = 0.013, 95% CI: 0 - 0.426, *p =* 0.015). Meanwhile, previous studies have shown that the free/total PSA ratio has higher diagnostic accuracy than the TPSA (19–21). In our study, the clinical significance of f/T PSA exceeded that of other PSA related indicators and was negatively associated with PCa. Thus, the f/T PSA was included in the prediction model according to the multivariable analysis.

Compared with blood, urine has the advantages of convenient material extraction and large sample size. Prostate Cancer Antigen 3 (PCA3) is a prostate cancer marker in the urine. In clinical applications, it has shown satisfactory results in PC detection, staging, and prognosis. According to the study, the specificity of PCA3 in the diagnosis of prostate cancer was 56.3-89%, and the sensitivity was 46.9-82.3% (2). PCA3 positivity was associated with high-grade PCa in the pathology (11). Furthermore, the amino acids and carnitine derivatives were significantly increased in the urine of patients with PCa, but hardly in most of the urine of patients with BPH. This provides a direction for the subsequent exploration of PCa related biomarkers (3). Besides, another predictor of prostate cancer, the SelectMDx score (MDx Health). It was determined by combining different levels of HOXC6 and DLX1 with clinical risk factors (age, DRE, PSA, PSAD, family history, previous negative biopsy) (7, 10). According to the study of Busetto GM et al., the selectMDx score predicted PCa on biopsy with a sensitivity of 94.1% and 91.4% specificity, which was significantly better than PSA (17.1%). In predicting csPCa, the sensitivity and the PSA were 100% identical, and the specificity of 73.3% was significantly better than the PSA (13.3%) (7). Another study on the SelectMDx scores showed that a sensitivity and specificity of 86.5% and 73.8% predicted PCa at biopsy, and 87.1% and 63.7% predicted csPCa at biopsy, respectively. The negative predictive value (NPV) for PCa and csPCa was 91.6% and 95.2%, respectively (10).

MRI is a crucial imaging technique for PCa diagnosis. It can be used in clinical practice for prostate cancer detection, biopsy, and monitoring of disease progression. And every patient requiring a biopsy is recommended for an MRI (11). Using mpMRI before prostate biopsy reduces unnecessary biopsies by 25% and may improve detection of csPCa (22). However, 10% to 20% of csPCa is not detectable by mpMRI (11). Therefore, additional predictors are needed to complement the MRI results and refine the decision-making on biopsy. Moreover, Current models combining radiomics and genomic biomarkers improve prostate cancer prediction power and have better applications. In addition, artificial intelligence and machine learning in PCa is also crucial for the diagnosis plays an important role (11, 12). In 2012, the European Society of Urogenital Radiology (ESUR) developed the PI-RADS v. 1 based on mpMRI to standardize image interpretation, reporting, and diagnosis of PCa. In 2015, the American College of Radiology (ACR), ESUR, and the AdMeTech Foundation revised and improved PI-RADS v. 1 to develop PI-RADS v. 2 (23). According to a previous meta-analysis on the diagnostic value of the PI-RADS v. 2 score for PCa. In this study, the sensitivity and specificity of the PI-RADS v. 2 score in diagnosing PCa were 89.0 and 73.0%, respectively. Compared to the PI-RADS v. 1, the PI-RADS v. 2 score was more accurate in detecting PCa (24). However, both malignant and benign prostate tumors have many similar mpMRI features (25). Hence, different levels of PI-RADS score only indicate the probability of tumor occurrence (23, 26). Herein, the PI-RADS score (OR = 3.356, 95% CI: 2.445-4.606, *p* < 0.001) was included as a risk factor in the prediction model.

The incidence of prostate cancer increases with age. A study on the prevalence of prostate cancer reported that the prevalence of prostate cancer was 5% in patients < 30 years and 59% in those > 79 years (27). Consistent with this conclusion, in our study, age (OR = 1.056, 95% CI: 1.022-1.092, *p* = 0.001) in the development cohort was also a significant risk factor for PCa. The age and PI-RADS score were significantly associated with adverse pathology (AP) at radical prostatectomy (RP) (28). At RP, AP was considered as non–organ-confined disease, and/or lymph node invasion and/or pathological grade group ≥ 3.

The current study shows that inflammatory actions are essential at different phases of tumor growth (29).Therefore, the blood inflammatory indicators were included in the research.Neutrophils are associated with the prognosis of many separate cancers (30) and are involved in almost all phases of tumor progression (31). Neutrophils also participate in lung cancer development (13) and breast cancer metastasis (14). Tumour-associated neutrophils have two opposing mechanisms and are classified according to their function into the N1-phenotype (anti-tumor effects) and the N2-phenotype (promotes tumourigenesis) (32). The N1-phenotype is regulated by TGF-β and can be converted to N2. TGF-β can be provided by the tumor or the tumor microenvironment (31, 32). Moreover, previous studies have shown that PCa patients have lower neutrophil count (33, 34), consistent with our current findings. The distribution of neutrophils was 3.89 ± 1.44 in the PCa group vs. 4.30 ± 1.67 in the non-PCa group for the development cohort (*p* = 0.005) and 3.83 ± 1.17 in the PCa group vs. 4.72 ± 2.08 in the non-PCa group (*p* < 0.001) for the validation cohort.

The HPR is calculated from hemoglobin and platelets, and its diagnostic and prognostic utility for tumors has been demonstrated (15, 16). Our current multivariable analysis showed that low HPR was a risk factor for PCa(OR = 0.136, 95% CI: 0.047- 0.417, *p<*0.001). Lower HGB levels have also been related to the development of colorectal cancer in previous studies (35). Herein, we showed that the two groups had different hemoglobin distributions (development cohort: 131.03 ± 20.49 vs. 135.96 ± 16.19, *p* = 0.004; validation cohort: 130.60 ± 17.53 vs. 141.04 ± 12.46, *p* < 0.001). Additionally, tumor patients with low hemoglobin had a worse prognosis (36, 37). Low hemoglobin levels can cause tumor hypoxia in cancer patients (38). Hypoxia in tumors can also cause alterations in the genetic code that contribute to tumor progression and aggressiveness (38, 39). Furthermore, an increased platelet count is a predictive factor for various tumors (40). Cancer patients with thrombocytosis have increased odds of adverse events. Besides, vascular embolism occurs in nearly 20% of cancer patients (41). Previous research has found that tumor cells cause thrombocytosis by boosting hepatic thrombopoietin (TPO) expression *via* IL-6 activation (42). Platelets can also accelerate tumor progression and invasion by producing cytokines, including vascular endothelial growth factor (VEGF), platelet-derived growth factor (PDGF), and transforming growth factor-β (TGF-β) (41, 42).

In this study, we included hematological indicators and clinical characteristics of patients to explore the risk factors of prostate cancer. Based on the results of the multivariate analysis for the development cohort, we included five risk factors associated with PCa development, including age (*p* = 0.001) and preoperative NEUT (*p* = 0.008), HPR (*p* < 0.001), f/T (*p* = 0.015), and PI-RADS score (*p* < 0.001). These factors included biological indicators and clinical information of patients. Therefore, we established a nomogram based on the relative risk of each factor to predict PCa probability, which was simple and convenient for clinical application. The internal validation of the nomogram showed that the C-index was 0.851, and the AUC was 0.851. The calibration curve presented great agreement. Herein, all indicators were collected from the patient before the biopsy. Thus, predicting patients’ probability of cancer with non-invasive approaches can be used to avoid unnecessary punctures and reduce pain.

However, our current study also had some limitations. First, this was a retrospective study with small sample size and might be subject to selection bias and interference by other uncharted factors. Second, patients’ data came from a single center, and the established predictive model was not externally validated. Therefore, the validity of this model needs to be tested in future studies. Despite these shortcomings, our findings demonstrated that combining patients’ biomarkers and clinical information could contribute to diagnosing PCa. Finally, our study population only included Chinese people, and the results of this study may not apply to other ethnic groups.

## Conclusion

In summary, we constructed a nomogram to predict PCa by integrating patients’ biological markers and clinical features. This nomogram provided a handy and non-invasive prostate cancer screening method for male.

## Data availability statement

The raw data supporting the conclusions of this article will be made available by the authors, without undue reservation.

## Ethics statement

The studies involving human participants were reviewed and approved by the ethics committee board of the First Affiliated Hospital of Wannan Medical College. Written informed consent for participation was not required for this study in accordance with the national legislation and the institutional requirements.

## Author contributions

WR, YX: data collection, data analysis, and manuscript writing. CY, PY, SF: data collection. LC, JH, DZ: project development and manuscript revision.

## Conflict of interest

The authors declare that the research was conducted in the absence of any commercial or financial relationships that could be construed as a potential conflict of interest.

## Publisher’s note

All claims expressed in this article are solely those of the authors and do not necessarily represent those of their affiliated organizations, or those of the publisher, the editors and the reviewers. Any product that may be evaluated in this article, or claim that may be made by its manufacturer, is not guaranteed or endorsed by the publisher.

## Abbreviations

AJCC, American Joint Committee on Cancer; AUC, Area under ROC curve; f/T, fPSA to TPSA ratio; HPR, Hemoglobin to platelet ratio; MLR, monocyte-to-lymphocyte ratio; NLR, neutrophil to lymphocyte ratio; PLR, platelet to lymphocyte ratio; PSA, Prostate-specific antigen; PV, Prostate volume; PSAD, PSA density; PI‐ RADS, Prostate Imaging-Reporting and Data System; ROC, receiver operating characteristic; SII, systemic immune-inflammation index.
