# Supplementary figures and images for: Development and validation of a predictive model for diagnosing prostate cancer after transperineal prostate biopsy
Source: Front Oncol. 2022 Dec 1;12:1038177. doi: 10.3389/fonc.2022.1038177 (PMC9751862; doi:10.3389/fonc.2022.1038177)

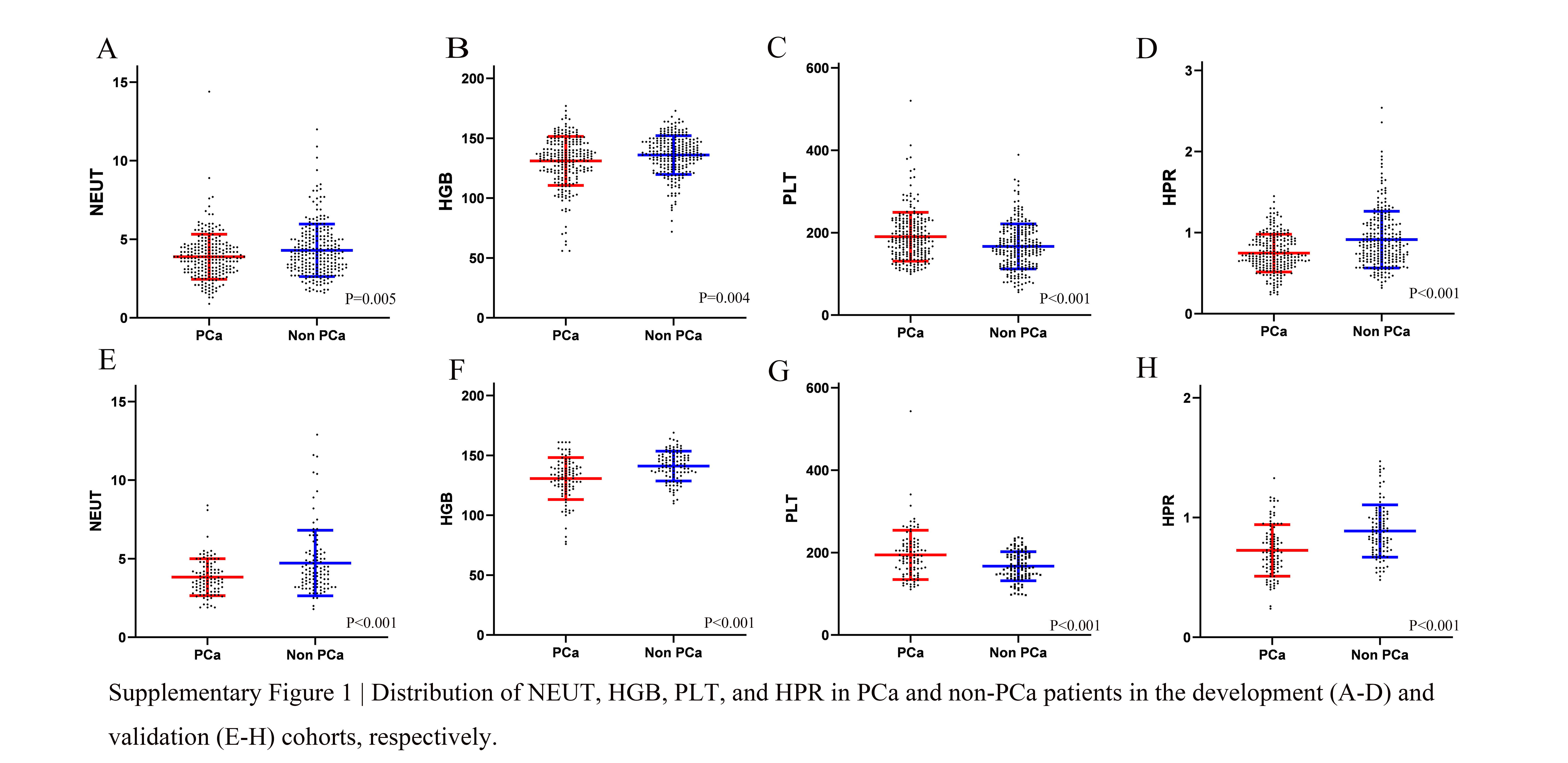

Supplement: Supplementary file 1 [file Image_1.jpeg]

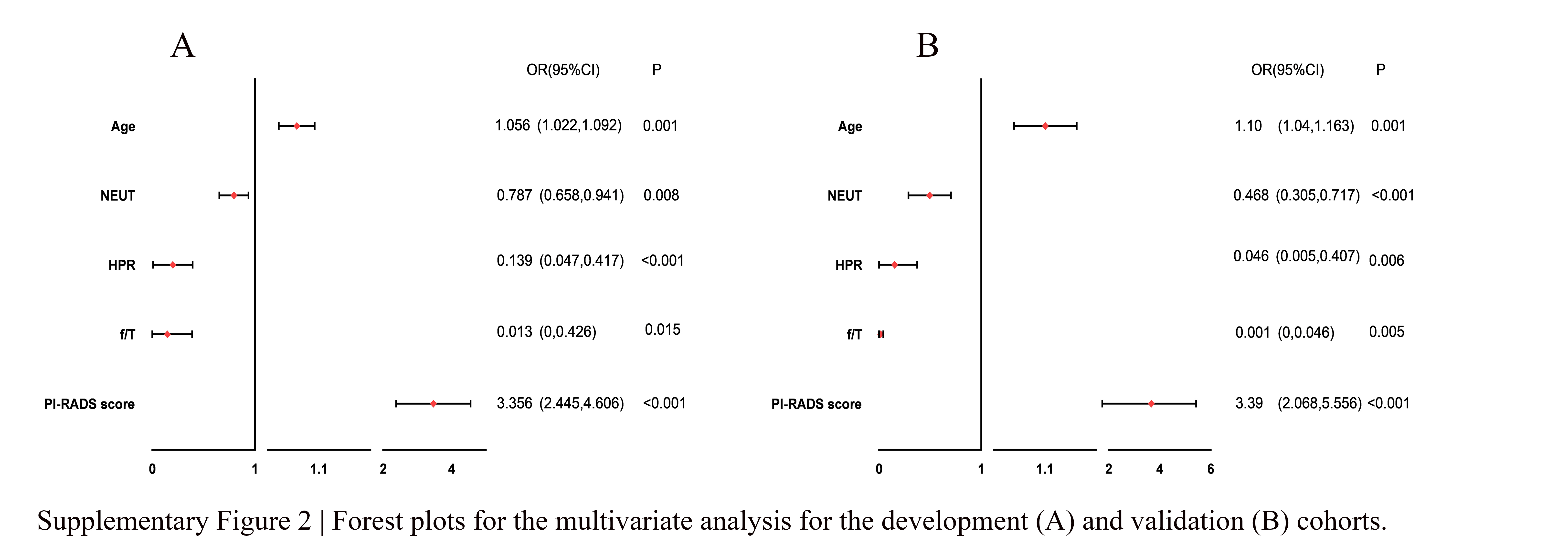

Supplement: Supplementary file 2 [file Image_2.jpeg]
